# Chemical characterization of iridoid glycosides from *Veronica persica* by UHPLC-MS/MS and their *in vitro* anti-inflammatory activity

**DOI:** 10.3389/fphar.2026.1871550

**Published:** 2026-07-17

**Authors:** Hanfeng Min, Lu Tan, Wenxin Li, Zhaoyang Wei, Kedi Yang, Qiuling Wang, Li Ge

**Affiliations:** 1 Medical School of Guangxi University, Nanning, China; 2 Yichang Humanwell Pharmaceutical Co., Ltd., Yichang, China; 3 Faculty of Chinese Medicine Science Guangxi University of Chinese Medicine, Nanning, China

**Keywords:** 6“-O-trans-feruloylcatalpol, anti-inflammatory activity, catalposide, iridoid glycosides, UHPLC-MS/MS, Veronica persica

## Abstract

Veronica persica: Poir., a common weed found along field ridges, has been traditionally employed in folk medicine for the treatment of severe burns. To assess the therapeutic effectiveness of *Veronica persica* against burns, a murine model of physical scald injury was established using a preheated metal plate. The results indicated that *V. persica* treatment significantly promoted wound healing, reducing the recovery period from 24 days (untreated controls) to 11 days (aqueous extract), comparable to the 10-day recovery observed with silver sulfadiazine ointment (positive control), while the ground plant material showed even faster healing in only 6 days. Using UHPLC-Q Exactive Orbitrap MS/MS, sixteen iridoid glycosides were successfully characterized, among which 6″-*O*-*trans*-feruloylcatalpol was detected in *V. persica* for the first time. Quantitative determination through UHPLC-Q Exactive Orbitrap MS/MS allowed relative quantification of five selected iridoid glycosides, yielding concentrations of verproside (0.12%), catalposide (0.72%), 6-*O*-veratroylcatalposide (0.16%), 6″-*O*-*trans*-feruloylcatalpol (0.0018%), and aucubin (0.27%). Additionally, these iridoid glycosides exhibited dose-dependent suppression of NO production and notably reduced the secretion of pro-inflammatory cytokines TNF-α and IL-1β in an LPS-stimulated RAW264.7 macrophage model. These results provide fundamental insights into the pharmacological mechanisms that may underlie the burn-healing activity of *V. persica*.

## 1 Introduction

*Veronica persica* Poir*.* is an annual herb of the genus *Veronica* (family Plantaginaceae) native to the Caucasus-northern Iran region and now widely distributed across temperate Eurasia (Fischer, 1987; Sarker et al., 2000). *Veronica persica* has been used in folk medicine to relieve rheumatic pain and knee soreness (Ishtiaq et al., 2024). In Chinese materia medica, this species is recorded as “Shenzicao,” and the whole plant is used medicinally for rheumatic arthralgia, chronic malaria, and related conditions. These sources also describe the external use of *V. persica* decoctions for scabies and other skin-related conditions (Jiangsu New Medical College, 1977; Zhonghua Bencao Editorial Committee, 1999). Other species of the genus *Veronica* have also been documented for topical use, including wound healing and the treatment of skin lesions (Salehi et al., 2019).

Multiple *Veronica* species have demonstrated diverse biological effects such as anti-inflammatory (Vogl et al., 2013), antibacterial (Stojković et al., 2013), antioxidant (Mocan et al., 2015), and anticancer activities (Živković et al., 2014). These pharmacological properties are generally attributed to abundant secondary metabolites, particularly iridoids, phenolic acids, and flavonoids. According to Gründemann (Gründemann et al., 2013) the anti-inflammatory efficacy of *Veronica officinalis* extracts might be linked to their significant content of iridoid glycosides. Additionally, iridoid compounds were isolated and identified independently from *Veronica cymbalaria* and *Veronica anagallis-aquatica* by Taskova (Taskova et al., 1999) and Su (Su et al., 2000), respectively.

Previous studies using inflammatory disease models have associated *V. persica* with anti-inflammatory and antioxidant activities. It has been shown that *V. persica* exerted anti-inflammatory effects (Fierascu et al., 2018). *Veronica persica* was also reported to alleviate acetaminophen-induced acute liver injury in mice by reducing oxidative stress and inflammatory responses, possibly through the NF-κB/STAT3, ERK/JNK, and AMPK signaling pathways (Tian et al., 2023). An ethanolic extract of *V. persica* further improved 2,4-dinitrochlorobenzene-induced atopic dermatitis-like skin inflammation. This effect was likely mediated by immune-response regulation and activation of the Nrf2/HO-1 pathway (Shim et al., 2023). These findings support the anti-inflammatory potential of *V. persica*. However, the constituents responsible for this activity remain unclear. Previous phytochemical studies have shown that *V. persica* contains various secondary metabolites. Iridoid glycosides represent the major class and include aucubin, catalpol derivatives, and verproside. Phenylethanoid glycosides, such as acteoside, isoacteoside, and plantamajoside, have also been identified and are considered major bioactive constituents (Harput et al., 2002). It has further been reported that *V. persica* is rich in iridoid glycosides, particularly glycosides with aucubin- and catalpol-type structures (Crişan et al., 2010). Iridoid glycosides have been associated with anti-inflammatory and antioxidant activities in plants. They have also been linked to pharmacological effects, including hepatoprotection and neuroprotection (Kumar et al., 2017; Wang et al., 2020; Zheng et al., 2025). However, the relationship between the biological activities and chemical composition of *V. persica* remains incompletely understood. Systematic characterization of its iridoid glycosides may help clarify the chemical basis underlying the traditional medicinal uses of this species.

UHPLC-Q Exactive Orbitrap MS/MS provides higher resolution, mass accuracy, sensitivity, and analytical speed than conventional HPLC-MS methods (Chen et al., 2018; Zheng et al., 2020). This technique allows the simultaneous qualitative and quantitative analysis of multiple metabolites and has been widely used for the comprehensive characterization of complex natural products. In a single analytical run, UHPLC-Q Exactive Orbitrap MS/MS can detect multiple compounds and distinguish structural isomers with high mass accuracy (Lin et al., 2024). By monitoring diagnostic precursor/product-ion signals, this method also enables rapid and sensitive quantification and provides reproducible responses suitable for trace-level analysis (Feng et al., 2023; Gong et al., 2025; Shao et al., 2025). For phytochemical studies, UHPLC-Q Exactive Orbitrap MS/MS is well suited for the comprehensive profiling and quantification of structurally diverse secondary metabolites (Zheleva-Dimitrova et al., 2023; Sun et al., 2023).

The LPS-induced RAW264.7 macrophage model is commonly used to evaluate inflammatory responses *in vitro*. In this model, NO, TNF-α, IL-1β, and IL-6 are used as standard inflammatory markers (Facchin et al., 2022). In this study, ultra-high-performance liquid chromatography coupled with Q-Exactive Orbitrap mass spectrometry (UHPLC-Q Exactive Orbitrap MS/MS) was used to identify and characterize iridoid glycosides in *V. persica*. Sixteen iridoid glycosides were identified in the aqueous extract by comparing retention times and mass spectral data with reference standards. Subsequently, semi-quantitative analysis was performed for five major iridoid glycosides that had been identified and isolated using conventional separation methods. These compounds were verproside, catalposide, 6-*O*-veratroylcatalposide, 6″-*O-trans*-feruloylcatalpol, and aucubin. Additionally, the anti-inflammatory potential of these five isolated compounds was evaluated using an *in vitro* cell model, providing experimental evidence to clarify the anti-inflammatory mechanisms of *V. persica*.

## 2 Materials and methods

### 2.1 Reagents and instruments

An ultrahigh-performance liquid chromatography system coupled with a quadrupole Exactive Orbitrap tandem mass spectrometer (UHPLC-Q Exactive Orbitrap MS/MS; Thermo Fisher Scientific) equipped with a heated electrospray ionization (HESI) interface was employed for both qualitative and quantitative assessments. Chromatographic separation was performed on an ACQUITY UPLCBEH C18 column (2.1 × 50 mm, 1.7 μm). Compound Discoverer 3.2 software (Thermo Fisher Scientific) was utilized to process the acquired raw data. Reference standards including verproside (≥98%), catalposide (≥98%), 6-O-veratroylcatalposide (≥98%), 6″-*O*-*trans*-feruloylcatalpol (≥98%), and aucubin (≥98%) were purchased from Shanghai Yuanye Biotechnology Co., Ltd. (Shanghai, China).

HPLC-grade methanol and formic acid (purity ≥99.9%) were purchased from Merck, located in Darmstadt, Germany. Ultrapure water was prepared with a MicroPure ultrapure water purification system (Thermo Fisher Scientific, Waltham, MA, USA). Analytical-grade solvents (purity ≥99%) were sourced from Guangdong Guanghua Sci-Tech Co., Ltd., Guangdong, China.

### 2.2 *Veronica persica* samples

Whole plants of *V. persica* Poir. (Plantaginaceae) were collected from Enshi, Hubei Province, China in April 2021. The plant material was authenticated by Prof. Hongqiang Zhang (Kunming Institute of Botany Biotechnology Co., Ltd., China). A voucher specimen (No. VP-2021041212) has been deposited in the Herbarium of Guangxi University School of Medicine, Guangxi, China. Plant material was ground into a fine powder using a mortar and pestle. Three extracts were prepared: absolute ethanol, 50% ethanol, and aqueous. Briefly, powdered material was refluxed separately with absolute ethanol or 50% ethanol (1:10, w/v) for 3 h. After filtration, solvents were removed under reduced pressure, yielding crude extracts. The aqueous extract was similarly prepared using deionized water (1:10, w/v) under the same conditions. Yields were 10.74% (aqueous), 18.22% (absolute ethanol), and 15.97% (50% ethanol), respectively. To ensure experimental reproducibility, the extraction and chemical characterization methods adhered to ConPhyMP/GA best-practice recommendations. According to ConPhyMP criteria, *V. persica* is neither a pharmacopoeial species nor widely used or commercially traded, categorizing the extract used in this study as extract type C. Completed ConPhyMP checklist tables 1 and 2c are provided as supplementary files.

### 2.3 Animals

Thirty-six male Kunming mice, 6–8 weeks of age, were obtained from the Animal Experiment Center of Guangxi University. Their initial body weight was 33.5 ± 1.2 g. The mice were housed in plastic cages under a 12 h light/dark cycle and were allowed free access to food and water throughout the experiment. All procedures involving animals were conducted in accordance with international ethical guidelines and were approved by the Guangxi University Institutional Animal Care and Use Committee (IACUC; approval no. GXU-2026-049; approved on 5 March 2026). Before burn induction, mice were anesthetized with isoflurane inhalation and received subcutaneous meloxicam (1–2 mg/kg) for analgesia. Throughout the study, pain-related indicators, including appearance, behavior, and body weight, were monitored.

### 2.4 Mouse treatment

Before the experiment, dorsal hair was removed, and the exposed skin was disinfected. Burn injury was induced under sterile conditions using a preheated metal plate (1 cm × 1 cm) at 100 °C. The plate was applied vertically to the skin surface for 10 s to create a deep dermal burn. The same device, temperature, contact time, and contact area were used for all animals. Burn severity was evaluated based on standardized induction parameters and gross morphological features; however, histopathological confirmation was not performed. Mice were randomly assigned to the following groups: a control group, *V. persica*-treated groups, and a positive control group treated with silver sulfadiazine cream. Each group contained six mice (n = 6).

### 2.5 Preparation of sample solutions and standard solutions for UHPLC-MS/MS

#### 2.5.1 Sample solution

Precisely 5.00 g of powdered *V. persica* was weighed and extracted with 50.00 mL deionized water by refluxing for 3 h. The extraction procedure was repeated thrice, and the combined extracts underwent concentration under reduced pressure via vacuum distillation. We dissolved the resulting extract in chromatography-grade methanol, sonicated the solution to facilitate dissolution, filtered it through a 0.22 μm membrane, and subjected the filtrate to qualitative analysis by UHPLC-Q Exactive Orbitrap MS/MS.

#### 2.5.2 Standard solution

We accurately weighed the reference standards, dissolved them in chromatography-grade methanol by ultrasonication, and prepared standard solutions at final concentrations of 0.01, 0.1, 0.3, 0.5, and 1 mg/L.

### 2.6 Qualitative analysis

Chromatographic separation occurred on an ACQUITY UPLC BEH C18 column (Waters, USA) with a maintained temperature of 30 °C, and the autosampler was set at 10 °C. Gradient elution involved a mobile phase of water containing 0.1% formic acid (solvent A) and methanol (solvent B). The gradient procedure was executed as follows: solvent A at 95% from 0 to 2 min; a gradual decrease from 95% to 0% A between 2 and 13 min; 0% A maintained from 13 to 16 min; solvent A quickly restored to 95% from 16 to 16.1 min; and finally, kept steady at 95% from 16.1 to 19 min. A flow rate of 0.3 mL/min was used, and the injection volume was 2 μL.

Mass spectrometric detection was conducted under positive and negative ionization conditions, with parameters including a spray voltage of 3.0 kV, HESI temperature set at 350 °C, and capillary temperature fixed at 320 °C. The sheath gas and auxiliary gas pressures were set to 35 psi and 10 psi, respectively, using nitrogen gas of high purity. Scanning was performed using Full MS/DD-MS2 mode, with a mass scan range from m/z 200 to 2000 and resolution values of 70,000 for initial scans and 17,500 for subsequent scans. Compound identification was carried out according to the FDA ORA LAB Manual (Section 5.4.5, 2023). Compounds were classified into three levels: Level 1, confirmed using reference standards; Level 2, based on MS/MS fragmentation patterns and literature comparison; and Level 3, tentative identification.

### 2.7 Semi-quantitative analysis

Semi-quantitative analysis was performed using UHPLC-Q Exactive Orbitrap MS in full-scan mode based on extracted ion chromatograms (EICs). The analysis of samples was conducted using UHPLC-Q Exactive (Thermo Fisher Scientific) under identical chromatographic and mass spectrometric conditions used for compound identification. External calibration curves were constructed using five reference standards at known concentrations. Linearity of each compound was verified, and quantification was performed by correlating the analyte peak areas in samples with corresponding calibration curves. This method was used for relative comparison of analyte abundance rather than absolute quantification. Therefore, full validation parameters, including LOQ, LOD, precision, and recovery, were not determined in this study.

### 2.8 Cytotoxicity assays

#### 2.8.1 Cell culture and processing

RAW264.7 murine macrophages were obtained from the Cell Bank of the Chinese Academy of Sciences (Shanghai, China).

The murine macrophage leukemia cell line RAW264.7 was maintained using DMEM medium enriched with 15% fetal bovine serum (FBS, volume ratio) and supplemented with 1% penicillin-streptomycin. Cells were grown at 37 °C within a humidified incubator containing 5% CO_2_.

#### 2.8.2 Cell viability assay

Before the anti-inflammatory assay, the cytotoxicity of the isolated monomer compounds (verproside, catalposide, 6-*O*-veratroylcatalposide, 6″-*O*-*trans*-feruloylcatalpol, and aucubin) against RAW264.7 cells was evaluated to confirm that their inhibitory effects on NO and cytokine production were not caused by reduced cell viability. Cell viability was evaluated via the CCK-8 assay (Zhang et al., 2022). RAW264.7 cells were plated at a density of 1 × 10^4^ cells/well into 96-well plates, with five replicates per group. After a 24 h incubation period at 37 °C, cells were further treated for another 24 h with varying concentrations (12.5, 25, 50, 100, and 200 μM) of isolated iridoid glycosides. Dexamethasone was evaluated at 1, 2.5, 5, 10, and 20 μM to confirm the non-cytotoxic concentration used as the positive anti-inflammatory control. Following treatment, 10 μL of CCK-8 reagent was added according to manufacturer guidelines, and plates were incubated for an additional 2 h at 37 °C. The optical density (OD) was measured at 450 nm using a microplate reader. The cell viability calculation was as follows: Cell viability (%) = [(OD_treatment_ − OD_blank_)/(OD_control_ − OD_blank_)] × 100%

### 2.9 *In vitro* anti-inflammatory activity assay

Cells from the RAW264.7 line were distributed into flat-bottomed 96-well microplates and allowed to adhere overnight. Subsequently, the cells were exposed to the test compounds at final concentrations of 12.5, 25, 50, and 100 μM and stimulated with LPS at a final concentration of 1 μg/mL for 24 h. Dexamethasone was included as the positive anti-inflammatory control in the NO and cytokine assays. Afterward, the culture medium from the macrophages was harvested for subsequent assays of NO, TNF-α, IL-6, and IL-1β. Concentrations of pro-inflammatory cytokines were assessed by ELISA, and nitric oxide levels were quantified utilizing the Griess assay.

### 2.10 Data processing and statistical analysis

All experiments were performed in triplicate and repeated in three independent experiments to ensure reproducibility. All results were expressed as means ± standard deviation values with triplicate determinations (*n* = 3). Data processing was performed using GraphPad Prism 8.0 (GraphPad Software, San Diego, CA, USA).

## 3 Results and discussion

### 3.1 Preliminary *in vivo* observation

To assess the therapeutic effect of *V. persica* on burn wound repair, macroscopic evaluation of wound models was conducted at different time points. The duration required for complete healing after burn induction was recorded and compared among groups.

**FIGURE 1:**
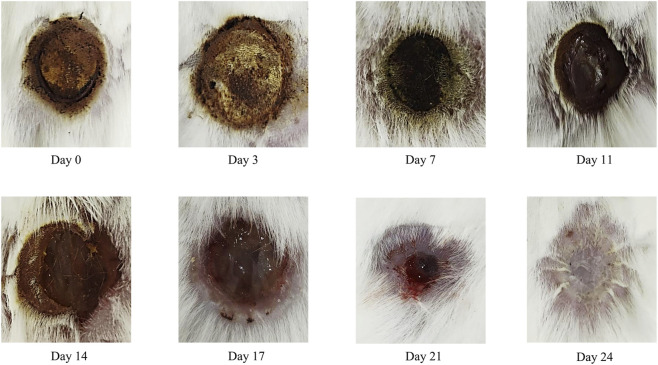
The wound healing process of the negative group (untreated) mice.

**FIGURE 2:**
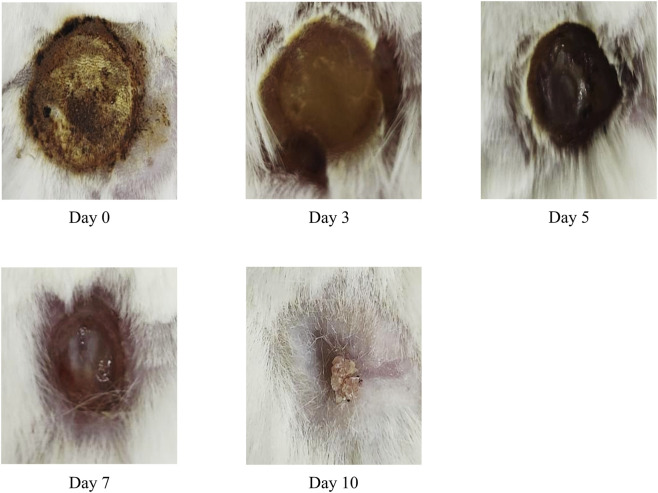
The wound healing process of the silver sulfadiazine cream group mice.

**FIGURE 3:**
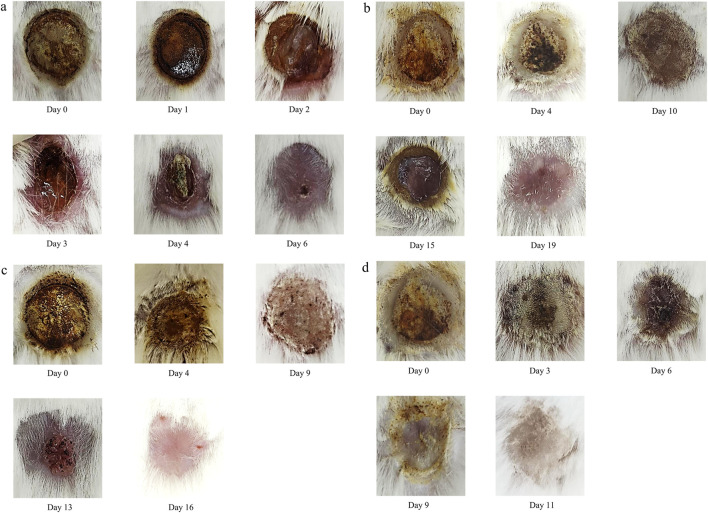
The wound healing process in mice treated with ground *Veronica persica* plant material **(a)**, 100% ethanol extract of *Veronica persica* **(b)**, 50% ethanol extract of *Veronica persica* **(c)**, and aqueous extract of *Veronica persica* **(d)**.

The findings indicated that *V. persica* treatment markedly reduced the healing period, although variations were observed among different extraction forms. In the negative control group (untreated group), complete wound closure required 24 days (Figure 1). In contrast, treatment with silver sulfadiazine cream (positive control) shortened the healing time to 10 days (Figure 2), confirming its therapeutic efficacy. The most prominent healing-promoting effect was observed in the ground plant material group (Figure 3a), where the healing time was reduced to 6 days, significantly better than the positive control group. The 100% ethanol extract (Figure 3b) and the 50% ethanol extract (Figure 3c) groups exhibited complete healing times of 19 and 16 days, respectively. The aqueous extract group (Figure 3d) had a healing time of 11 days, which was slightly longer than that of the ground group but still demonstrated a strong promoting effect similar to the positive control (Figure 2).

Among the experimental groups, the ground plant material group showed the strongest wound-healing effect. This result suggests that *V. persica* contains bioactive constituents that may promote wound repair, possibly through anti-inflammatory, antioxidant, and antibacterial activities. The healing time of the aqueous extract group was slightly longer than that of the ground plant material group. However, compared with the positive control, its wound-healing effect remained meaningful for further investigation. This finding suggests that water-soluble constituents may contribute to accelerated wound repair.

Wound healing critically depends on the inflammatory reaction, which is a precisely orchestrated physiological mechanism. This process aids in tissue regeneration by clearing microbial pathogens, dead cells, and debris, as well as recruiting and stimulating fibroblast activity. However, excessive or prolonged inflammation may impair the healing process. It can result in sustained tissue injury, oxidative stress, delayed repair, and eventually chronic wounds or hypertrophic scar formation (Ibrahim et al., 2018). During burn wound healing in mice, *V. persica* may mainly promote tissue repair through its anti-inflammatory activity. This effect may attenuate excessive inflammation, reduce tissue damage, shorten repair time, and promote orderly wound resolution. In the animal experiments, three extracts, namely, ethanol extract, 50% ethanol extract, and aqueous extract, were systematically evaluated. Among them, the aqueous extract showed superior therapeutic efficacy compared with the other two extracts. Aqueous extracts generally contain polar, water-soluble constituents. Therefore, this result suggests that water-soluble components may be involved in the effect of *V. persica* on wound healing. Previous studies have shown that *V. persica* contains iridoid glycosides and phenylethanoid glycosides. These compounds are relatively polar and may be enriched in aqueous extracts. Both iridoid glycosides and phenylethanoid glycosides have also been reported to exhibit anti-inflammatory and antioxidant activities. Therefore, the relatively favorable wound-healing effect of the aqueous extract of *V. persica* may be related to its enriched polar active components and their regulatory effects on inflammation and oxidative stress. Accordingly, the aqueous extract was selected for further phytochemical characterization and comprehensive analysis.

It should be noted that, in this study, wound healing was evaluated mainly by visual observation of wound closure time. No quantitative analysis of wound area, calculation of wound closure rate, or statistical comparison between groups was performed. These results should therefore be interpreted as preliminary observations rather than conclusive evidence of the wound-healing efficacy of *V. persica*. Future studies should include these quantitative assessments, together with histological evaluation, to fully validate its wound-healing efficacy.

### 3.2 Characterization of iridoids in *Veronica persica* by UHPLC-Q Exactive Orbitrap MS/MS

In the present study, metabolomics analysis was designed as a targeted approach because the primary aim was to characterize selected known or expected constituents related to biological activity, rather than to comprehensively discover unknown metabolites. Targeted analysis was chosen to improve the specificity and reliability of annotation for these predefined compounds. Because the aqueous extract may also contain metabolites outside the predefined target list, this targeted approach cannot fully describe its metabolite profile. Future studies will include untargeted LC-MS/MS analysis to explore additional metabolites in the aqueous extract.

**FIGURE 4:**
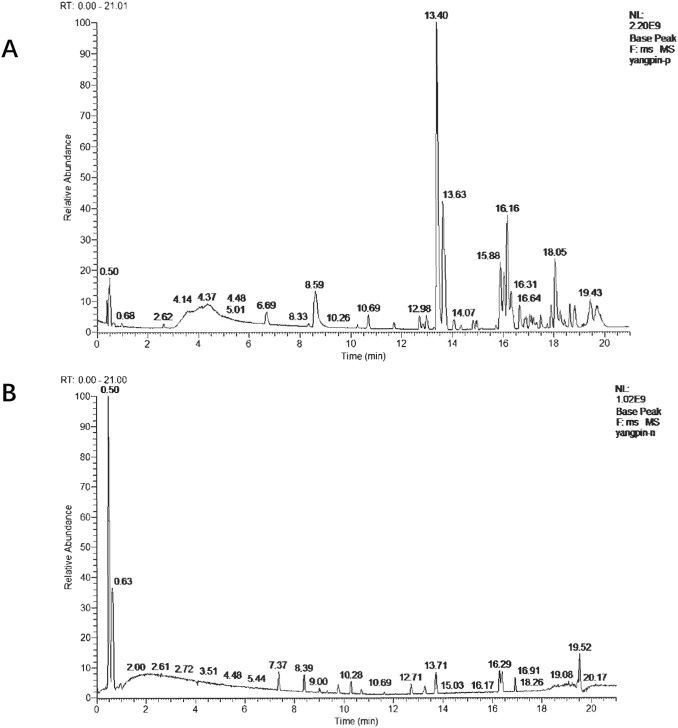
Base peak ion chromatogram of *Veronica persica* extract in positive **(A)** and negative **(B)** modes obtained by UHPLC-Q-Exactive Orbitrap MS/MS.

Qualitative profiling of the aqueous extract of *V. persica* was performed using UHPLC-Q Exactive Orbitrap MS/MS, resulting in the identification of 16 iridoid glycosides. Base peak chromatograms were obtained in both negative and positive ionization modes (Figure 4). Retention times and corresponding MS data are shown in Table 1. Some isomeric iridoids, such as loganic acid and mussaenosidic acid, could not be unambiguously distinguished under the present LC-MS conditions and were therefore annotated as putative isomers.

**TABLE 1:** Characterization of iridoids in *Veronica persica* by UHPLC–Orbitrap MS/MS.

NO.	Compound	Formula	*t*_*R*_ */*min	Observed	MS^n^	Level
1	Scutellarioside II	C_24_H_28_O_12_	7.97	507.14970 (M-H^-^)	163.03876,145.02818, 119.04876	Level 2
2	Picroside II	C_23_H_28_O_13_	8.52	511.14461 (M-H^-^)	349.09171,235.06065, 167.03381	Level 2
3	Verproside	C_22_H_26_O_13_	7.29	497.12964 (M-H^-^)	335.07535,221.04456, 153.01793	Level 1
4	Agnuside	C_22_H_26_O_11_	7.16	465.13913 (M-H^-^)	303.07243,121.02792, 137.02324	Level 2
5	Veronicoside	C_22_H_26_O_11_	10.38	467.15478 (M + H^+^)	305.03383,119.04922, 165.05481	Level 2
6	Geniposidic acid	C_16_H_22_O_10_	3.88	373.11292 (M-H^-^)	211.06020,193.04926, 167.07024, 149.05949	Level 2
7	(+)-Genipin	C_11_H_14_O_5_	10.4	225.07575 (M-H^-^)	207.10197,147.19839	Level 2
8	Bartsioside	C_15_H_22_O_8_	13.2	329.12309 (M-H^-^)	311.22165, 269.15302	Level 2
9	Aucubin	C_15_H_22_O_9_	19.53	345.11800 (M-H^-^)	183.06544,165.0548, 139.03902, 121.02848	Level 1
10	Monotropein	C_16_H_22_O_11_	4.79	389.10783 (M-H^-^)	227.01763, 209.04678	Level 2
11	Loganic acid/Mussaenosidic acid	C_16_H_24_O_10_	4.88	375.12857 (M-H^-^)	213.07625,169.08583, 151.07527	Level 3
12	Catalposide	C_22_H_26_O_12_	8.38	481.13405 (M-H^-^)	205.04968,177.05464, 137.02307	Level 1
13	PicrosideIII	C_25_H_30_O_13_	8.12	537.16026 (M-H^-^)	337.09299,193.04958, 175.03880,160.01523, 132.02026	Level 2
14	6-*O*-veratroylcatalposide	C_24_H_30_O_13_	16.86	525.16028 (M-H^-^)	363.10211,213.07656, 183.05521, 167.04503	Level 1
15	6″-*O-trans*-feruloylcatalpol	C_25_H_30_O_13_	16.19	537.16029 (M-H^-^)	337.09311,193.04927, 175.03899, 160.01541	Level 1

All identified constituents belonged to, or were structurally derived from, iridoids. Iridoid glycosides are structurally distinctive monoterpenoid natural products characterized by a cyclopentopyran core and commonly occur as *β-D*-glucosides in nature. Structural diversity arises from modifications of the parent nucleus, including cyclopentane ring cleavage (forming *seco*-iridoids), substitution with hydroxyl or ester groups, and variation in glycosylation sites and sugar types. These structural differences determine both chemical classification and pharmacological activity.

Previous studies have indicated that the anti-inflammatory and antioxidant activities of iridoid glycosides are related to their structural stability, glycosidic bonds, and acyl substituent types (Viljoen et al., 2012). The stronger anti-inflammatory and antioxidant effects of verproside (Peak 3) may be attributed to the two free phenolic hydroxyl groups in its substituents. Agnuside (Peak 4) is an aromatic acylated derivative formed by p-hydroxybenzoyl esterification of the C-10 hydroxymethyl group of aucubin. This substituent introduces an aromatic ring and phenolic hydroxyl groups. These groups may alter lipid solubility, hydrogen-bonding capacity, and molecular conformation. They may thereby affect molecular interactions with inflammation-related targets or signaling pathways. Previous studies have shown that agnuside can downregulate PGE2, LTB4, and various inflammatory cytokines, suggesting that its anti-inflammatory activity may be related to its aromatic acylated structure (Pandey et al., 2012). Veronicoside (Peak 5) also contains an aromatic fragment. However, it lacks a phenolic hydroxyl group on the benzoyl moiety, and its antioxidant activity is weaker than that of similar compounds containing phenolic hydroxyl groups or caffeoyl substituents. This finding indicates that the number and substitution pattern of phenolic hydroxyl groups on the aromatic acyl group may be important structural factors affecting activity differences in this class of compounds (Yin et al., 2016). These results suggest that, even among iridoid glycosides with the same or similar core skeletons, pharmacological activity may differ according to the type of substituent and the pattern of structural modification.

The iridoid skeleton typically consists of a cyclopentane ring fused with a hemiacetal structure. During MS/MS fragmentation, characteristic neutral losses are commonly observed, including H_2_O, CH_3_OH, and glucose (Glc). These losses generate fragment ions such as [M-H-Glc-H_2_O-CO_2_]^-^, and [M-H-Glc]^-^. Compounds including Picroside II (Peak 2), and geniposidic acid (Peak 6) are classified as seco-iridoids. These compounds are derived from cleavage of the C7-C8 bond in the iridoid core, forming a cyclopentane-fused pyran structure. In addition to common neutral losses, characteristic bond cleavages within the pyran ring were observed during collision-induced dissociation (Li et al., 2017).

**FIGURE 5:**
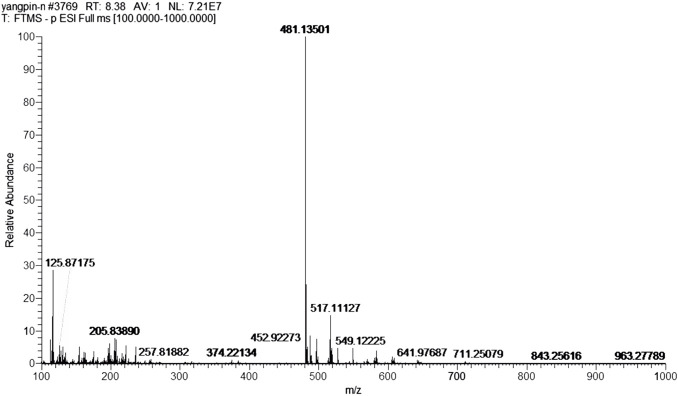
Product ion spectra of catalposide in the negative ion mode.

**FIGURE 6:**
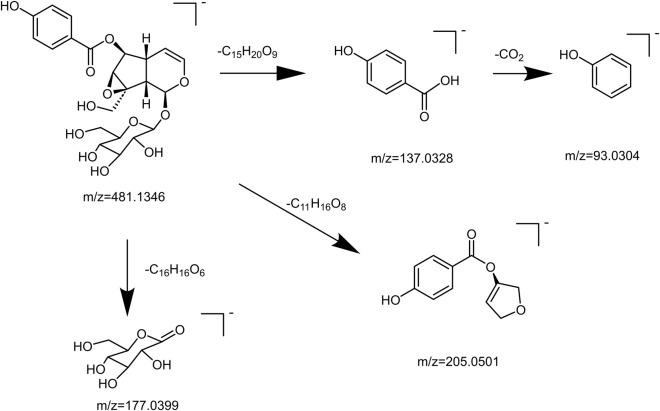
Proposed fragmentation pathways of catalposide under negative-ion mode.

Cyclopentanoid-type iridoids contain a *cis*-fused dihydropyran ring and a five-membered ring. The hemiacetal moiety may undergo isomerization, leading to fragmentation of the dihydropyran ring (Wu et al., 2013). For example, catalposide (Peak 13) exhibited a representative fragmentation pattern in the negative ion mode consistent with these structural features (Figures 5, 6).

The precursor ion m/z 481.13498[M-H]^-^ (C_22_H_26_O_12_) glycosidic bond breaking and ring opening generate fragments m/z 177.0399 (C_6_H_10_O_6_), m/z 205.0498 (C_11_H_10_O_4_) and m/z 137.0238 (C_7_H_6_O_3_), respectively, and the fragment m/z 137.0238 (C_7_H_6_O_3_) sheds 1 molecule of CO_2_ and finally forms a deprotonated molecular ion m/z 93.03307 (C_6_H_6_O).

### 3.3 Semi-quantitative analysis of five compounds in *Veronica persica* by UHPLC-Q Exactive Orbitrap MS/MS

Based on qualitative identification results, UHPLC-Q Exactive Orbitrap MS/MS was further employed for semi-quantitative analysis. Five major iridoid glycosides in *V. persica* were determined based on external calibration curves. Regression equations, correlation coefficients (*R* ^*2*^), and linear ranges (10–500 ng/mL for all analytes) are presented in Table 2. Since the analytical method was not fully validated for absolute quantification, these values should be interpreted as estimated relative contents.

**TABLE 2:** Regression equations, correlation coefficients, and contents of five compounds in *Veronica persica*.

NO.	Compound	Calibration curve	*R* ^*2*^	Content (%)
1	Verproside	*y* = 188699.31*x*+ 5500167.70	0.9791	0.1204
2	Catalposide	*y* = 291325.34*x* + 6345336.61	0.9845	0.7171
3	6-*O*-veratroylcatalposide	*y* = 199.50*x* + 861.56	0.9998	0.1592
4	6″-*O-trans*-feruloylcatalpol	*y* = 34364.31*x* + 515904.52	0.9871	0.0,018
5	Aucubin	*y* = 1473.1*x* + 9284.3	0.9998	0.2686

Semi-quantitative results (Table 2) demonstrated that catalposide was the predominant constituent (0.7171%), representing the major iridoid glycoside in the plant. Aucubin (0.2686%) and 6-*O*-veratroylcatalposide (0.1592%) were also present at relatively high levels. Verproside was detected at a lower concentration (0.1204%), whereas 6″-*O*-*trans*-feruloylcatalpol (0.0018%) was the least abundant compound.

These findings reveal substantial variation in the distribution of iridoid glycosides in *V. persica*. Catalposide was the dominant constituent and may contribute substantially to the pharmacological activity of the plant. Catalposide and other catalpol-type iridoid glycosides appeared to be relatively abundant in these samples. Previous studies have reported anti-inflammatory activity for catalpol-type iridoid glycosides, aucubin, and verproside. Therefore, the presence of these compounds may partly explain the chemical basis of the anti-inflammatory activity of *V. persica*. However, the semi-quantitative content of an individual compound cannot directly establish its contribution to the pharmacological activity of the plant. Low-abundance constituents, such as 6″-*O-trans*-feruloylcatalpol, may also exhibit activity or contribute to the overall effect through synergistic interactions. Further clarification of the contribution of each compound will require validated quantitative methods combined with activity-guided evaluation. This compositional diversity provides a valuable basis for further pharmacological research and rational utilization of *V. persica* resources.

### 3.4 Cytotoxicity

**FIGURE 7:**
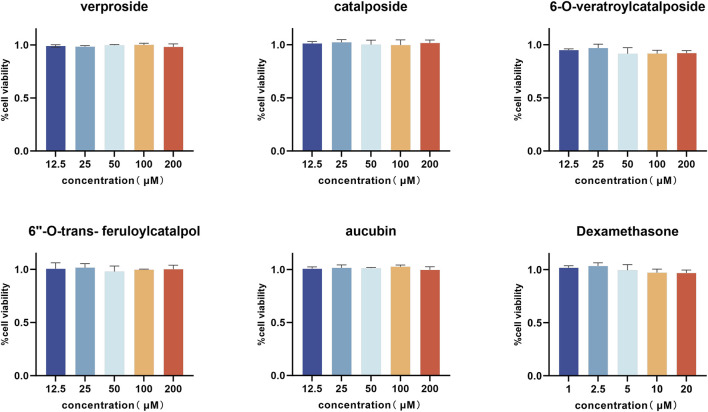
Percentage cell viability of verproside (1), catalposide (2), 6-*O*-veratroylcatalposide (3), 6″-*O*-trans-feruloylcatalpol (4), aucubin (5) in RAW 264.7 cells at concentrations of 12.5, 25, 50, 100 and 200 µM after 24 h of treatment. Percentage cell viability of dexamethasone at concentrations of 1, 2.5, 5, 10, 20 µM. (mean ± SD, n = 3)

As shown in Figure 7, RAW264.7 cells were treated with compounds 1-5 at final concentrations of 12.5, 25, 50, 100, and 200 μM for 24 h. The cell survival rates in all compound-treated groups remained above 90% within the range of 12.5–100 μM, indicating no evident cytotoxicity within this concentration range. At the highest concentration of 200 μM, most compound-treated groups still maintained high cell viability, although compound 2 caused a slight decrease to 92%. These results suggest that compounds 1-5 showed low cytotoxicity under the tested conditions and were suitable for further pharmacological evaluation.

### 3.5 Inhibition of NO levels in LPS-induced RAW264.7 cells by *Veronica persica*

To evaluate the effects of the isolated compounds on NO production, RAW264.7 cells were stimulated with LPS (final concentration, 1 μg/mL) and treated with compounds 1-5 at 12.5, 25, 50, and 100 μM for 24 h.

A standard curve was generated using NaNO_2_ solutions at gradient concentrations to quantify nitric oxide levels in the culture supernatant. The strong linear correlation between nitrite concentration and absorbance at 540 nm (*R* ^2^ = 0.9999) confirmed the reliability of the Griess assay. Based on the cytotoxicity results, suitable concentrations for *in vitro* determination (12.5, 25, 50, and 100 μM) were selected. The molar concentration required to achieve 50% inhibition relative to the control group (IC_50_) was calculated and is shown in Table 3. During co-incubation with LPS, the positive control dex showed a strong inhibitory effect on NO production (IC_50_ = 1.9 ± 0.7 μM). Compounds 1-3 significantly inhibited nitric oxide production in a clear dose-dependent manner. Compound 1 showed the strongest inhibitory effect (IC_50_ = 61.2 ± 2.8 μM), followed by compound 3 (IC_50_ = 86.6 ± 3.2 μM) and compound 2 (IC_50_ = 94.5 ± 4.6 μM). The inhibitory activities of compounds 4 and 5 were relatively weak (IC_50_ > 100 μM). These results suggest that specific iridoids in *V. persica* can suppress LPS-induced NO overproduction in macrophages.

**TABLE 3:** The molar concentration (μM) giving 50% inhibition (IC_50_) relative to the vehicle control. (mean ± SD, *n* = 3).

NO.	Compound	IC_50_(μM)
1	Verproside	61.2 ± 2.8
2	Catalposide	94.5 ± 4.6
3	6-*O*-veratroylcatalposide	86.6 ± 3.2
4	6″*-O-trans*-feruloylcatalpol	>100
5	Aucubin	>100
6	Dexamethasone (positive control)	1.9 ± 0.7

### 3.6 Effect of *Veronica persica* on LPS-induced release of TNF-α, IL-1β, and IL-6 in RAW 264.7

**FIGURE 8:**
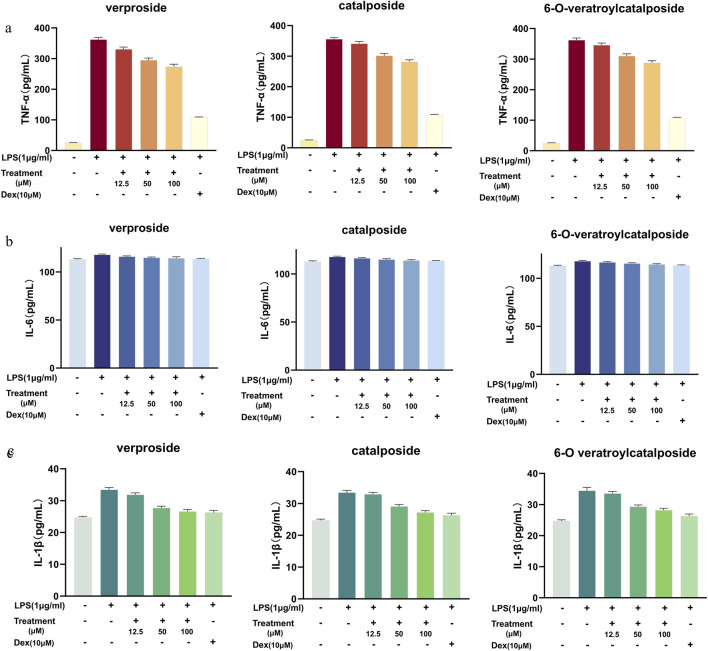
Effect of verproside, catalposide and 6-*O*-veratroylcatalposide on the production of TNF-α **(a)**, IL-6 **(b)** and IL-1β **(c)** in LPS-induced RAW 264.7 cells. Dexamethasone (10 μM) was used as the positive control.

As illustrated in Figure 8, treatment with verproside, catalposide, and 6-*O*-veratroylcatalposide reduced the secretion of TNF-α and IL-1β in LPS-stimulated RAW264.7 cells, with dexamethasone (10 μM) included as the positive control. No statistically significant inhibition of IL-6 secretion was observed at the tested concentrations. Among these compounds, verproside showed the strongest concentration-dependent inhibition of TNF-α and IL-1β release. These findings suggest that the isolated iridoid glycosides may attenuate LPS-induced inflammatory responses, which is consistent with previous reports on the anti-inflammatory effects of iridoid glycosides in LPS-stimulated RAW264.7 cells (Moncada and Bolaños, 2006). In the inflammatory response, cytokines and inflammatory mediators, such as nitric oxide (NO), are significantly increased. Excessive production of inflammatory mediators may amplify the inflammatory response and further cause tissue damage (Zheng et al., 2025). In this study, the Griess assay showed that verproside exhibited the strongest inhibitory effect on LPS-induced NO production. After macrophages are activated by LPS, substantial NO production is usually associated with increased expression of iNOS. Therefore, the iridoid glycosides in *V. persica* may inhibit NO production by regulating the expression or activity of iNOS.

At the molecular level, NO generation and cytokine release are usually regulated by inflammation-related signaling pathways, such as MAPK and NF-κB. Studies have shown that some natural compounds can reduce the expression of inflammation-related genes by affecting these signaling pathways, thereby alleviating inflammatory responses (Beg et al., 2011; Rosillo et al., 2014; Medicherla et al., 2016). Although this study did not directly detect the activation status of the NF-κB or MAPK signaling pathways, the observed decreases in NO, TNF-α, and IL-1β suggest that iridoid glycosides in *V. persica* may regulate macrophage inflammatory responses by affecting both NO production and cytokine release. The specific molecular mechanism requires further study and verification. Overall, the results of this study indicate that some iridoid compounds in *V. persica* have *in vitro* anti-inflammatory effects. Further studies are needed to determine whether these compounds have potential as natural anti-inflammatory active components.

## 4 Conclusion

This study characterized the iridoid glycoside profile of *V. persica* Poir. using UHPLC-Q Exactive Orbitrap MS/MS. Sixteen iridoid glycosides were identified, and five major compounds, namely, verproside, catalposide, 6-*O*-veratroylcatalposide, 6″-*O*-*trans*-feruloylcatalpol, and aucubin, were further analyzed semi-quantitatively. Biological evaluation showed that some of these isolated iridoid glycosides suppressed LPS-induced production of the pro-inflammatory cytokines TNF-α and IL-1β, as well as the inflammatory mediator NO, in RAW264.7 macrophages. The compounds showed no evident cytotoxicity within the tested concentration range. Their anti-inflammatory activity may be associated with the regulation of iNOS expression and related inflammatory signaling pathways.

## Data Availability

The datasets presented in this study can be found in online repositories. The names of the repository/repositories and accession number(s) can be found in the article/Supplementary Material.
